# The thickness of the ventral medial prefrontal cortex predicts the prior-entry effect for allocentric representation in near space

**DOI:** 10.1038/s41598-022-09837-y

**Published:** 2022-04-05

**Authors:** Jie Huang, Aijun Wang, Xiaoyu Tang, Ming Zhang

**Affiliations:** 1 Department of Psychology, Research Center for Psychology and Behavioral Sciences, Soochow University, Suzhou, People’s Republic of China; 2 School of Psychology, Liaoning Collaborative Innovation Center of Children and Adolescents Healthy Personality Assessment and Cultivation, Liaoning Normal University, Dalian, People’s Republic of China; 3 Cognitive Neuroscience Laboratory, Graduate School of Interdisciplinary Science and Engineering in Health Systems, Okayama University, Okayama, Japan

**Keywords:** Cognitive neuroscience, Social neuroscience

## Abstract

Neuropsychological studies have demonstrated that the preferential processing of near-space and egocentric representation is associated with the self-prioritization effect (SPE). However, relatively little is known concerning whether the SPE is superior to the representation of egocentric frames or near-space processing in the interaction between spatial reference frames and spatial domains. The present study adopted the variant of the shape-label matching task (i.e., color-label) to establish an SPE, combined with a spatial reference frame judgment task, to examine how the SPE leads to preferential processing of near-space or egocentric representations. Surface-based morphometry analysis was also adopted to extract the cortical thickness of the ventral medial prefrontal cortex (vmPFC) to examine whether it could predict differences in the SPE at the behavioral level. The results showed a significant SPE, manifested as the response of self-associated color being faster than that of stranger-associated color. Additionally, the SPE showed a preference for near-space processing, followed by egocentric representation. More importantly, the thickness of the vmPFC could predict the difference in the SPE on reference frames, particularly in the left frontal pole cortex and bilateral rostral anterior cingulate cortex. These findings indicated that the SPE showed a prior entry effect for information at the spatial level relative to the reference frame level, providing evidence to support the structural significance of the self-processing region.

## Introduction

The ability to represent surrounding spatial information is an essential requirement of living beings because individuals must represent spatial orientation information at all times in the real three-dimensional world. Generally, different spatial representation strategies have been used to represent spatial orientation information as quickly and accurately as possible^1^, and it has been suggested that individuals use either egocentric (i.e., subject-to-object relations to form body-centered representations) or allocentric (i.e., object-to-object relations to form world-centered representations) reference frames to represent information^2,3^. However, individuals had a different preference weight for the frame of reference. Evidence from behavioral and neuropsychological studies has supported that individual preferentially adopt different reference frames to represent spatial information in different depth locations^4–7^.

Previous studies have suggested that the space domain is divided into near (within the range of the arm) and far (beyond the range of the arm) spaces based on the hand-reaching distance^5,8,9^. According to the perception/action model, the dorsal stream transforms visual information into sensorimotor representation and the ventral stream transforms visual information into perceptual representation^10,11^. Therefore, the dorsal stream is implicated in near-space processing when individuals conduct an action and manipulation representation, and the ventral stream is implicated in far-space processing when individuals conduct a perception representation^4,7,10,11^. However, extant studies suggested that the differential activations in the dorsal/ventral stream associated with near space and far space processing were independent of the type of task but related to the frame of reference^4,7,12–15^. Evidence from clinical studies has shown that egocentric representation is usually transformed into the corresponding sensorimotor representation through the dorsal stream and the allocentric representation is usually transformed into the corresponding perceptual representation through the ventral stream^14–19^. For instance, it has been proved that patient with a damaged ventral stream had a poor performance on an allocentric task; likewise, a patient who injured the dorsal stream had a poor performance on an egocentric task^4,7,19–21^. Based on the perception/action model, egocentric representation and near-space shared a common neural mechanism (i.e., dorsal stream); similarly, allocentric representation and far-space shared common a neural mechanism (i.e., dorsal stream). Therefore, theoretically, individuals are prone to use egocentric references in near space and allocentric references in far space.

Nevertheless, studies on the interaction between spatial domains and reference frames have found that individuals show processing precedence for egocentric presentation and near-space processing^4,6,7,22^. For example, Chen and colleagues^4^ found that participants preferred egocentric representation, with a faster response in egocentric tasks regardless of near or far space. Additionally, the fMRI results by Chen and colleagues revealed that the parietal-occipital junction (POJ) shows enhanced neural activity in near-space processing, indicating a near-space preference. The study of Wang et al.^7^ further supported the view that the POJ acts as an interface between the dorsal and ventral streams in near and far space processing, showing higher activity to the target in near space than in far space. Therefore, individuals actually showed a priority for near-space processing and egocentric representation when reference frames interacted with spatial domains.

Because objects that unexpectedly approach the observer have higher self-relevancy and farther unexpected objects involve less self-related thoughts, previous studies have indicated that attentional reorientation along 3D space involves self-related processing^23–26^. Briefly, the objects in the near space could automatically attract attention compared to those in far space^27–30^. For instance, Cosman and Vecera^27^ implied that individuals took precedence to process objects in near space because of the processing order determined by attention. Therefore, due to the high attention priority and strong self-relevancy of objects in near space, individuals tend to show a self-prioritization effect (SPE) in near-space processing. SPE is the phenomenon in which individuals focus on self-related information or objects and respond faster (or more accurately) relative to information associated with others^31–34^, such as the self-face advantage effect^35^ and self-reference effect^36–39^.

Currently, converging studies have established the SPE using a shape-label matching task^40–51^ and found a faster response of self-association pairings than stranger-association pairings and higher activation of the ventral medial prefrontal cortex (vmPFC) in self-referential processing^33,38,52–55^. Existing studies suggested that the self gave priority not only to near-space processing but also to egocentric representation^18,41,42,44^. Specifically, participants tended to adopt an egocentric bias (self-reference bias) to represent internal representation, as reflected in the priority of the SPE for egocentric representation^4,44,54,55^, which might be because individuals were prone to perceive scenes from a first-person perspective^56,57^. It was suggested that taking an embodied perspective (i.e., first-person perspective) could enhance the self-bias in perceptual matching^43^. In addition, from the view of the cognitive map, the flow of allocentric representation through ownership to the self (medial temporal lobe, temporal cortex, lateral parietal cortex, and medial prefrontal cortex, respectively) requires more brain regions than that of egocentric representation (from the lateral parietal lobe to the medial prefrontal cortex)^2^. Altogether, the self had a higher priority for the information of near-space processing and egocentric representation.

Considering the role of the SPE in near space processing and egocentric representation, the present study was specifically designed to address how the SPE controls the interaction between spatial domains and spatial reference frames. Specifically, participants addressed the conflict between performing allocentric visuospatial judgments in near space and egocentric visuospatial judgments in far space. Addressing this issue would allow a better understanding of the priority of the SPE and clarify the role of the SPE within the real 3D spatial domain. In the present study, all the participants were randomly assigned to either the self-association group or stranger-association group, and both groups were required to complete the spatial reference frame task in near and far spaces. The self-association group only needed to respond to the self-associated object, while the stranger-association group only needed to respond to the stranger-associated object. To further investigate whether the cortical thickness of self-processing regions could predict the SPE on spatial reference frames, we set the vmPFC as a region of interest (ROI) and extracted the cortical thickness of the vmPFC using surface-based morphometry analysis to correlate with the performance in the spatial reference frame task. We hypothesized that the SPE could affect the performance of egocentric representation and near-space processing in the self-association group and showed a preference for the process of the object in near space, followed by egocentric representation. Additionally, the cortical thickness of the vmPFC might be correlated with the performance of near-space processing and egocentric representation.

## Results

### Behavioral data

To ensure that the samples were representative and unbiased, nine participants were excluded from the statistical analysis because they had low accuracy (lower than 90%) in the spatial reference frame judgment task, possibly because of the lack of attention or motivation during the experiment. Because the overall accuracy of all the participants was high (all above 94%), the present study focused more on reaction time (RT) in the statistical analysis (see Table 1).

**Table 1:** Mean reaction times (ms) and standard deviations (ms) for all conditions between the self-association group and the stranger-association group.

	Self-association group		Stranger-association group	
	Egocentric	Allocentric	Egocentric	Allocentric
Near	587 (86)	571 (73)	617 (90)	595 (77)
Far	685 (100)	636 (78)	613 (73)	645 (91)

**Figure 1:**
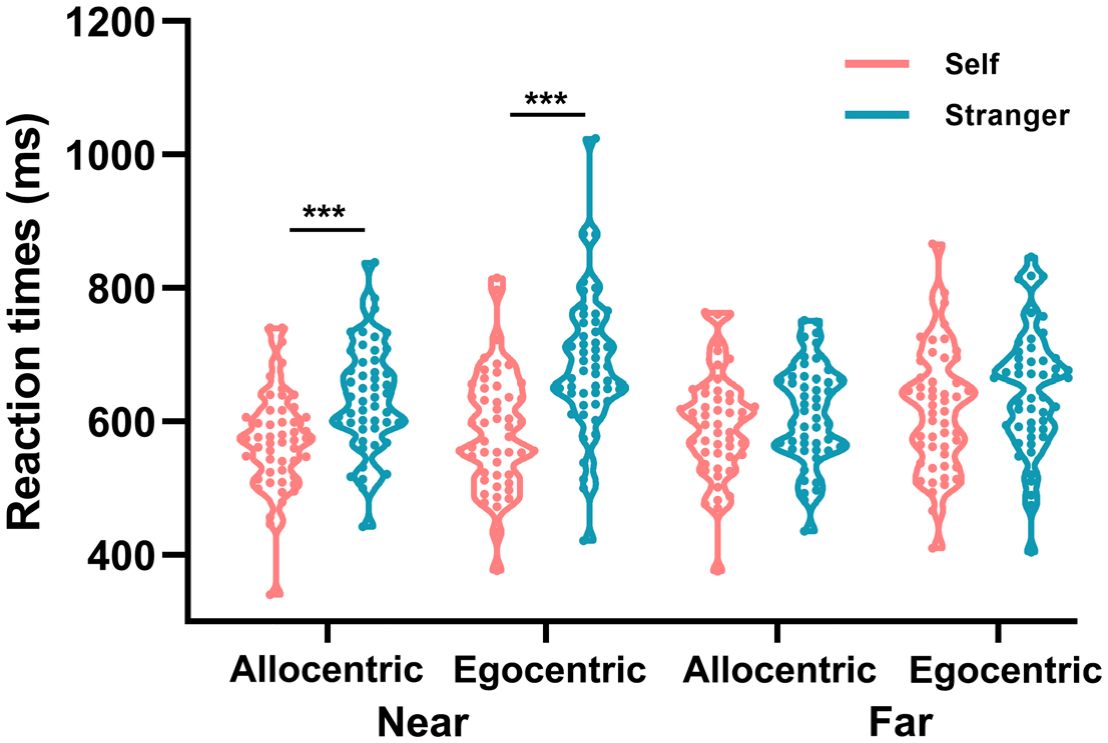
Mean reaction times in spatial reference frame tasks. The RT of the self-association group was faster than that of the stranger-association group (*p* = 0.001). Additionally, the RT of the allocentric judgment task in near space (571 ms) was faster than that of the egocentric judgment task in far space (617 ms) in the self-association group (*p* < 0.001). The RT of the allocentric judgment task was faster than that of the egocentric judgment task (*p* < 0.001). A significant interaction effect was found among groups, spatial domains, and reference frame judgment tasks. Specifically, in near space, the reaction times of allocentric representation in the self-association group (571 ms) were faster than those in the stranger-association group (636 ms) (*p* < 0.001), and the reaction times of egocentric judgment tasks in the self-association group (587 ms) were faster than those in the stranger-association group (685 ms) (*p* < 0.001). No difference was found in egocentric or allocentric judgment tasks between the groups in far space (*ps* > 0.05).

For the RT data, 2 (groups: self-association vs. stranger-association) × 2 (spatial domains: near vs. far) × 2 (reference frame judgment tasks: allocentric vs. egocentric) mixed ANOVA was conducted (see Fig. 1). The main effect of groups was significant, *F*(1,97) = 11.10, *p* = 0.001, *η*_*p*_^2^ = 0.10, and the RTs of the self-association group (592 ms) were faster than those of the stranger-association group (645 ms), indicating a significant SPE in the self-association group. The main effect of spatial domains was not significant, *F* < 1. The main effect of reference frame judgment tasks was significant, *F*(1,97) = 35.02, *p* < 0.001, *η*_*p*_^2^ = 0.27, and the RTs of the allocentric judgment task (604 ms) were significantly faster than those of the egocentric judgment task (633 ms). The interaction effect between groups and spatial domains was significant, *F*(1,97) = 61.70, *p* < 0.001, *η*_*p*_^2^ = 0.39. The interaction effect between groups and reference frame judgment tasks was significant, *F*(1,97) = 4.89, *p* = 0.03, *η*_*p*_^2^ = 0.05. The interaction effect between the spatial domains and reference frame judgment tasks was not significant, *F*(1,97) = 1.12, *p* = 0.29. The three-way interaction effect among groups, spatial domains, and reference frame judgment tasks was significant, *F*(1,97) = 4.59, *p* = 0.04, *η*_*p*_^2^ = 0.05. To investigate the potential interaction between ownership groups and reference frames in near and far spaces, further simple effect analyses should be conducted.

First, to examine the role of groups in the three-way interaction effect, 2 (spatial domains: near vs. far) × 2 (reference frame judgment tasks: allocentric vs. egocentric) repeated ANOVA was conducted in the self-association group and stranger-association group. For the self-association group, the main effect of spatial domains was significant, *F*(1,49) = 25.57, *p* < 0.001, *η*_*p*_^2^ = 0.33, and the RTs of near-space processing (579 ms) were significantly faster than those of far-space processing (606 ms). The main effect of reference frame judgment tasks was significant, *F*(1,49) = 6.35, *p* = 0.02, *η*_*p*_^2^ = 0.12, and the RTs of the allocentric judgment task (583 ms) were significantly faster than those of the egocentric judgment task (602 ms). The interaction effect between spatial domains and reference frame judgment tasks was not significant, *F* < 1. To examine whether the participants performed better in allocentric judgment tasks in near space or egocentric judgment tasks in far space, a paired sample *t* test was conducted in the self-association group. The RTs of the allocentric judgment task in near space (571 ms) were faster than those of the egocentric judgment task in far space (617 ms), *t*(50) = 5.02, *p* < 0.001, *Cohen’s d* = 0.56. For the stranger-association group, the main effect of spatial domains was significant, *F*(1,48) = 38.62, *p* < 0.001, *η*_*p*_^2^ = 0.45, and the RTs of near-space processing (629 ms) were faster than those of far-space processing (660 ms). The main effect of reference frame judgment tasks was significant, *F*(1,48) = 36.16, *p* < 0.001, *η*_*p*_^2^ = 0.43, and the RTs of the allocentric judgment task (624 ms) were faster than those of the egocentric judgment task (665 ms). The interaction effect between spatial domains and reference frame judgment tasks was significant, *F*(1,48) = 4.24, *p* < 0.045, *η*_*p*_^2^ = 0.08. Further analysis found that the RTs of the allocentric judgment task (636 ms) were faster than those of the egocentric judgment task (685 ms) in far space, *F*(1,48) = 34.41, *p* < 0.001, *η*_*p*_^2^ = 0.42, and the RTs of the allocentric judgment task (613 ms) were also faster than those of the egocentric judgment task (645 ms) in near space, *F*(1,48) = 19.66, *p* < 0.001, *η*_*p*_^2^ = 0.29, exhibiting faster allocentric processing. These findings indicated that individuals showed a preference for allocentric representation in near space compared with egocentric representation in far space.

Second, to examine the role of reference frame judgment tasks in the three-way interaction effect, a 2 (groups: self-association vs. stranger-association) × 2 (spatial domains: near vs. far) mixed ANOVA was conducted in the egocentric and allocentric judgment tasks. For the egocentric judgment task, the main effect of groups was significant, *F*(1,97) = 12.57, *p* = 0.001, *η*_*p*_^2^ = 0.12, and the RTs of the self-association group (602 ms) were significantly faster than those of the stranger-association group (665 ms). The main effect of spatial domains was not significant, *F* < 1. The interaction effect between groups and spatial domains was significant, *F*(1,97) = 41.37, *p* < 0.001, *η*_*p*_^2^ = 0.30. Further analysis found that the RTs of the self-association group in near space (587 ms) were faster than those of the stranger-association group (685 ms), *F*(1,97) = 26.68, *p* < 0.001, *η*_*p*_^2^ = 0.22, and no significant difference was found in the RTs between the self-association (617 ms) and stranger-association (645 ms) groups in far space. For the allocentric judgment task, the main effect of groups was significant, *F*(1,97) = 7.61, *p* = 0.007, *η*_*p*_^2^ = 0.07, and the RTs of the self-association group (583 ms) were significantly faster than those of the stranger-association group (624 ms). The main effect of spatial domains was not significant, *F* < 1. The interaction effect between groups and spatial domains was significant, *F*(1,97) = 49.42, *p* < 0.001, *η*_*p*_^2^ = 0.34. Further analysis found that the RTs of the self-association group in near space (571 ms) were faster than those of the stranger-association group (636 ms), *F*(1,97) = 18.06, *p* < 0.001, *η*_*p*_^2^ = 0.16, and no significant difference was found in the RTs between the self-association (595 ms) and stranger-association (613 ms) groups in far space. The above findings revealed that the SPE strongly affected near-space processing compared with far-space processing.

Third, to examine the role of spatial domains in the three-way interaction effect, a 2 (groups: self-association vs. stranger-association) × 2 (reference frame judgment tasks: allocentric vs. egocentric) mixed ANOVA was conducted in near and far spaces. In the far space condition, the main effect of groups was not significant, *F*(1,97) = 2.02, *p* = 0.16. The main effect of reference frame judgment tasks was significant, *F*(1,97) = 27.12, *p* < 0.001, *η*_*p*_^2^ = 0.22, and the RTs of the allocentric judgment tasks (604 ms) were significantly slower than those of the egocentric judgment tasks (631 ms). The interaction effect between groups and reference frame judgment tasks was not significant, *F*(1,97) = 1.17, *p* = 0.28. In the near space condition, the main effect of groups was significant, *F*(1,97) = 25.46, *p* < 0.001, *η*_*p*_^2^ = 0.21, and the RTs of the self-association group (579 ms) were significantly faster than those of the stranger-association group (660 ms). The main effect of reference frame judgment tasks was significant, *F*(1,97) = 28.65, *p* < 0.001, *η*_*p*_^2^ = 0.23, and the RTs of the allocentric judgment task (603 ms) were significantly faster than those of the egocentric judgment task (636 ms). The interaction effect between groups and reference frame judgment tasks was significant, *F*(1,97) = 7.50, *p* = 0.007, *η*_*p*_^2^ = 0.07. Further simple effect analysis showed that the RTs of the egocentric judgment task (685 ms) were slower than those of the allocentric judgment task (636 ms) in the stranger-association group, *t*(48) = 5.87, *p* < 0.001, *Cohen’s d* = 0.54. However, no significant difference was found between allocentric (571 ms) and egocentric (587 ms) judgment tasks in the self-association group, *t*(49) = 1.81, *p* = 0.08. To further examine whether the SPE was more beneficial for egocentric representation or allocentric representation, we obtained the reaction time difference (RT_allocentric-egocentric_) by comparing the RT difference between allocentric and egocentric judgment tasks in near space. The independent-sample *t* test results showed a significant difference between the self-association group and stranger-association group, *t*(97) = 2.74, *p* = 0.007, *Cohen’s d* = 0.55, and the RT difference in the self-association group (16 ms) was less than that in the stranger-association group (49 ms), indicating that the self-association group had a faster response on the egocentric judgment task in near space. These findings suggested that the SPE only benefited egocentric representation more than allocentric representation.

### sMRI correlation analysis

As discussed in the Introduction section, the vmPFC region is supported by self-referential material^33,38,52–55^. Additionally, the behavioral results benefited the SPE more on the allocentric reference frame in near space than on the egocentric reference frame in far space. Therefore, we focused on examining whether the cortical thickness of the vmPFC (i.e., OFC, ACC, FPC, and insular cortex) could predict the preference of the SPE on reference frames.

**Figure 2:**
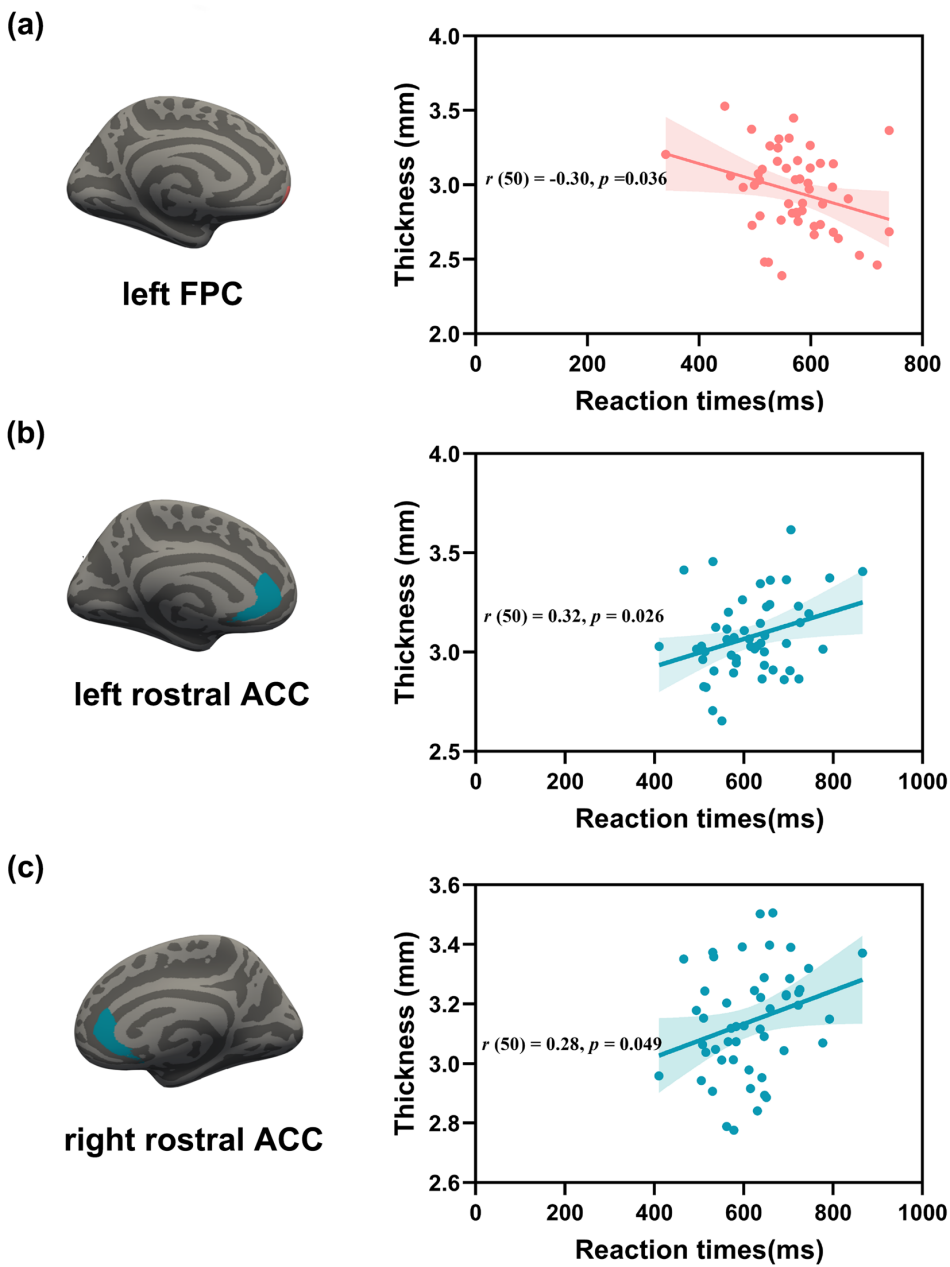
Behavioral SPE-related cortical thickness of ROIs. (**a**) The RTs of the allocentric judgment task in near space were negatively related to the thickness of the left frontal pole cortex (*p* = 0.036). (**b**) The RTs of the egocentric judgment task in far space were positively related to the thickness of the left rostral anterior cingulate cortex (*p* = 0.026). (**c**) The RTs of the egocentric judgment task in far space were positively related to the thickness of the right rostral anterior cingulate cortex (*p* = 0.049).

**Figure 3:**
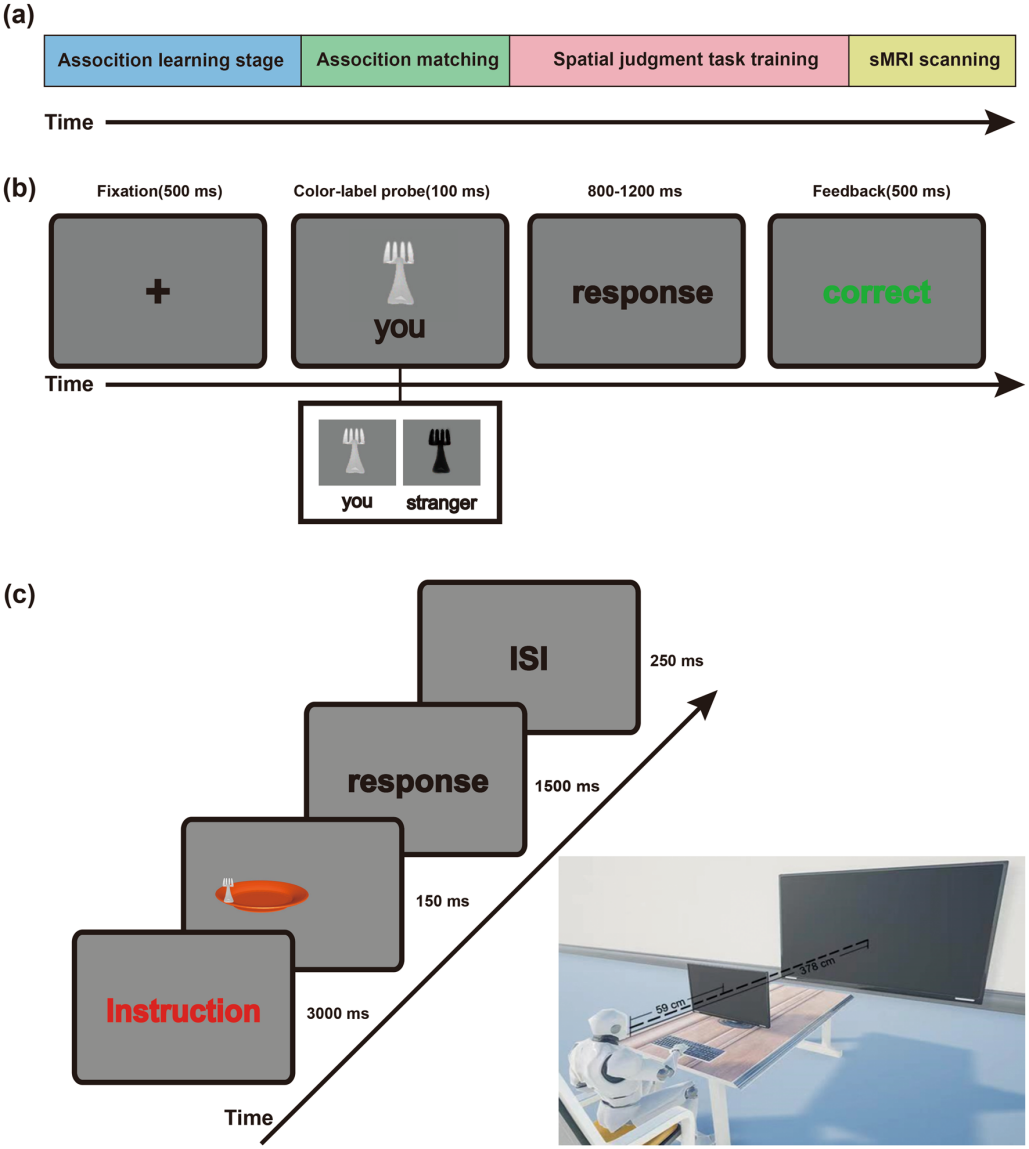
(**a**) Flowchart of the whole experiment. The overall experimental procedure comprised an association learning stage, an association training stage, the spatial reference frame judgment task, and structural MRI scanning. (**b**) Example stimuli and time of the single trial of the color-label matching task. The association between the color (black or white) and the label (you or stranger) was bound in the association learning stage. During the training stage, each trial started with a 500 ms duration fixation, followed by a probe stimulus for 100 ms. The participants had to determine whether the color-label pairing matched as soon as possible within the timeframe ranging from 800 to 1200 ms. (**c**) Procedure and temporal profile of stimulus presentation in the spatial reference judgment task. This task was presented block-by-block. Each block started with text guidance to inform participants of the type of task in the current block. The participants had to judge whether the fork was on the left or right side of the midline of their body in the egocentric judgment task and judge whether the fork was on the left or right side of the midline of the plate in the allocentric judgment task. Each block started with the presentation of instructions for 3000 ms. The probe stimulus (a colored fork intersecting an orange plate) was presented for 150 ms afterward. The participants were asked to judge the position of the fork according to the instructions as accurately and quickly as possible within 1500 ms. The duration of each trial was 1650 ms with a 250 ms interstimulus interval.

Pearson correlation analysis was conducted between the RTs of allocentric judgment tasks in near space and thickness of the vmPFC. Only the thickness of the left FPC was negatively associated with the RTs of the allocentric judgment task (see Fig. 2a), *r*(50) = − 0.30, *p* = 0.036; other regions showed little or no significant associations with RTs of the allocentric judgment tasks (*ps* > 0.05). Likewise, Pearson correlation analysis showed that the thickness of the left rostral ACC was positively correlated with the RTs of egocentric judgment tasks in far space (see Fig. 2b), *r*(50) = 0.32, *p* = 0.026; the thickness of the right rostral ACC was also positively correlated with the RTs of egocentric judgment tasks in far space (see Fig. 3c), *r*(50) = 0.28, *p* = 0.049. A significant association was no longer found between the thickness and RTs of the egocentric judgment task (*ps* > 0.05).

## Discussion

The present study used the color-label matching paradigm to establish an SPE and combined a conjunction visual search task to probe how the SPE influenced the judgment of the reference frames in different spatial domains. Participants were randomly divided into the self-association group and stranger-association group. The self-association group was required to respond to the object associated with themselves, while the stranger-association group was required to respond to the object associated with strangers. As expected, a significant SPE was found in the self-association group. Besides, the SPE showed preference processing for the allocentric reference frame in near-space compared with the egocentric reference frame in far space, revealing a preference for near-space processing, followed by egocentric representation. More importantly, correlation analysis showed a significant correlation between the cortical thickness of the vmPFC and RTs of reference frame tasks in near and far spaces, indicating that the thickness of the vmPFC could predict the difference in the SPE on reference frames.

First, the present study found a robust SPE in the self-association group compared with the stranger-association group. More specifically, the RTs of the spatial reference frame judgment task in the self-association group (592 ms) were significantly faster than those in the stranger-association group (645 ms). In accordance with the present study, previous studies have demonstrated that self-relevance automatically enhances stimulus processing, particularly in the form of a shape-label matching paradigm^36,40,45,46^. One point to note here is that the present study modified the associative-learning task and adopted the color-label matching task to familiarize participants and inform them about a stable color-label association, an approach that was different from the classic shape-label matching paradigm^40–42^. Yin and colleagues^45^ first adopted this novel color-label matching paradigm to establish a self-/friend-/stranger-association in a spatial working memory task, and participants responded faster to the working memory probes of self-associated colors than to those of friend-associated and stranger-associated colors. Recently, their following study replicated the SPE in the matching paradigm^46^. Thus, these findings suggest that the SPE in the self-association matching paradigm works at a conceptual level, regardless of the attribution of associated stimuli^47^.

Second, consistent with the findings of previous studies of a close relationship among the SPE, near-space processing, and egocentric reference frame^4,27–30,41,42^, the present study found that the SPE affected the interaction between spatial reference frames and spatial domains. More importantly, the SPE showed a preference for information of the allocentric reference frame in near space compared with the egocentric reference frame in far space. Specifically, the performance of the allocentric judgment task in near space (571 ms) was faster than that of the egocentric judgment task in far space (617 ms) in the self-association group, with performance exhibiting faster RTs. Moreover, further analysis suggested that the SPE was only observed in near-space processing, particularly in egocentric judgment tasks. Specifically, the performance of near-space processing (579 ms) was faster than that of far-space processing (606 ms) in the self-association group, and improvement of the SPE on the egocentric reference frame (16 ms) was better than that on the allocentric reference frame (49 ms) in near space. Therefore, we observed that the SPE benefited egocentric representation more based on near space, a finding that has not previously been described^18,44^. In other words, near-space precedence was observed compared with egocentric representation during the information processing between spatial-level and reference frame-level information, revealing a near prior entry effect.

Here, we believe that at least three factors should be considered for this consequence. In the first place, the attention priority to objects in near space may explain this consequence. Although the self-associated stimuli assigned increased personal significance and became perceptually more salient^41^, developing an egocentric bias to represent objects in daily lives revealed that self-related objects (information) captured more endogenous attention than unfamiliar objects^18,28,45^. The participants focused more on the self-related stimuli cues when they adopted the egocentric strategy in the egocentric reference frame judgment task. However, the processing of information in near space still automatically captures more attention than egocentric representation information. Many current views have posited that objects in near space tend to attract extensive attention and be processed preferentially, and objects in near space acquire high attentional priority and automatically draw exogenous attention^26–30^. For example, Abrams et al.^28^ argued that individuals tend to process objects in near space or those close to them because objects in near space automatically capture their attention, revealing a near prior entry effect. Furthermore, from the perspective of evolutionary psychology, the memory and visuospatial attention of humans are selective^58^. Focusing on stimuli in near space is important for an organism to survive and thrive, and individuals generally tend to focus on information or objects closely related to adaptability^58,59^, likely because information in near space is more urgent and threatening to individuals. In summarize, individuals had higher attention priority in the processing of near space than in egocentric representation.

Next, the common neural mechanism between the SPE and near-space processing may be the other possible underlying consideration. Evidence from neuropsychological studies has implied that the POJ, a neural interface for integrating and processing different kinds of information, is significantly activated when the egocentric reference frame interacts with far space and the allocentric reference frame interacts with near space^4,7,25,26^. Moreover, the POJ showed enhanced neural activity in near-space processing^4,7,22^. Furthermore, because of the urgency and immediacy of objects in near space, near-space processing showed high self-relevancy^23–26^. Chen et al. found that the default-mode network, including the posterior cingulate cortex, orbital prefrontal cortex, and left angular gyrus, showed higher activation of attentional orientation when participants completed the task in near space^48^. Given that the region of the POJ overlapped with self-processing regions, a common neural mechanism likely exists between the SPE and near-space processing. Thus, the SPE having a prior entry effect in near space processing is not surprising.

Afterwards, the execution of arm-movement responses may be the last consideration. Previous studies have found a performance advantage in matching the self label with an action representation^34,47^ but have not measured the action movement time. To further measure the response execution and examine whether response execution can be modulated by self-associations, Desebrock and colleagues^60^ combined a motor-variant shape-label paradigm with a response box to measure the reaction time and movement time of perceptual tasks and found that the SPE could modulate the response execution. Because near space has been related to action more than far space^10–13^, the arm-movement response was actually an action representation^60,61^. That is why the SPE showed more priority to the processing of near space.

Third, regarding correlation analysis, our results supported the view that the SPE was associated with the cortical midline structure, particularly the vmPFC^33,38,52–55,62^. Specifically, the thickness of the left FPC was negatively correlated with the RTs of the allocentric judgment task in near space, and the thickness of the bilateral rostral ACC was positively correlated with the RTs of the egocentric judgment task in far space, a finding that contrasted the closed relationship between the SPE and ACC. Cortical thickness measures whether the brain structure is damaged or reveals normal development in morphological analysis of brain structure^63–66^. Better development of the brain area is generally associated with a higher cortical thickness. For example, Fleming et al. indicated that the thicker the right anterior cingulate cortex was, the better the cognitive control performance^64^. Because of the dominance of spatial-level information processing, it is not surprising that the thicker the FPC is, the faster the response of the allocentric reference frame in near space. However, notably, the positive correlation in the present study may be due to processing conflict between the information of the spatial level and reference frame level. Many studies have demonstrated that vmPFC activity (such as the ACC) is involved in various self-processing tasks^38,51–53^. The present study found superiority in information processing at the spatial level compared with the reference frame level. In other words, our brains prioritized processing far space information and then processed the information of the egocentric reference frame when the egocentric judgment task was conducted in far space. Therefore, an individual with a thicker ACC will have stronger competition (i.e., take more cost) in the information processing between far-space processing and egocentric representation, exhibiting slower RTs with thicker thickness.

In conclusion, the present study revealed that the SPE could modulate the interaction between spatial domains and spatial reference frame representation. Additionally, the information at the spatial level before the reference frame level manifested with near-space processing first, followed by egocentric representation. Furthermore, the cortical thickness of the left FPC and bilateral ACC could predict the difference in the SPE on the reference frame. These findings reveal the role of the SPE in reference frames in the real three-dimensional world and provide evidence to support the relationship between the vmPFC and the SPE in humans regarding brain structure.

## Methods

### Participants

A total of 108 undergraduate and graduate students (42 male; age: 21.13 ± 1.95 years) participated in the experiment. The participants were randomly assigned to the self-association group and stranger-association group, with fifty-four participants in each group. All the participants were right-handed and had normal hearing, vision (or corrected vision), and color vision, with no history of neurological or psychiatric disorders. None of the participants had participated in any similar experiment. The present study was conducted according to the Declaration of Helsinki and was approved by the Ethics Committee of Soochow University. All the participants provided written informed consent. The sample size was calculated using the G-Power 3.1 toolbox^67,68^. According to a previous study^69^, a hybrid design should have a medium effect size (f = 0.25). With α = 0.05 and power = 0.80, the appropriate sample size was calculated to be at least 82. Nine participants were excluded from the statistical analysis because of lower accuracy.

### Apparatus and materials

The behavioral experiment was conducted on a laptop computer (Lenovo ThinkPad E480) with a screen resolution of 1024 × 768 pixels and a refresh rate of 60 Hz. The experiment comprised a delayed match-to-sample task (i.e., color-label matching) and a spatial reference frame task (Fig. 3a). The color-label matching task was a modified version of the shape-label association task used by Sui et al.^40^. During the color-label matching task, the visual stimuli were presented on a laptop screen with a gray background (RGB: 125, 125, 125) in E-prime 2.0 software. The visual stimuli comprised colored forks (e.g., black and white) and social labels (e.g., you and stranger). The black fork (RGB: 0, 0, 0) and white fork (RGB: 255, 255, 255) were presented above a fixation cross (0.8° × 0.8°) at the center of the screen (see Fig. 3b). The word “you” or “stranger” (3.1°/1.6°) was displayed below the fixation cross. The distance between the center of the color or word and the fixation cross was 3.5°.

The spatial reference frame judgment task was a modified version of the virtual spatial reference frame judgment task used by Chen et al.^4^. The spatial reference frame judgment task involved two parts. The first part was a reference frame judgment task (i.e., egocentric and allocentric) in near space. The second part was a reference frame judgment task in the far space. During the spatial reference frame judgment task (Fig. 3c), the stimuli in near space were presented on the laptop screen with a gray background (RGB: 125, 125, 125) in Presentation software (Neurobehavioral Systems Inc.), and the stimuli in the far space were presented on an EPSON CB-X29 projector under the same conditions. The visual stimuli comprised a colored fork (black or white) with a radius angle of 2.5° intersecting an orange plate (RGB: 220, 75, 30) with a radius angle of 15°. The fork could appear at one of four egocentric positions. For each of the four egocentric locations of the fork (− 5°, − 3.5°, 3.5°, or 5°), the location of the plate varied independently around the fork, using four possible allocentric positions (− 2.4°, − 1.7°, 1.7°, or 2.4°). The monitor (projector) was viewed at distances of 59 cm and 3.78 m in the near- and far-space tasks, respectively. The visual angles of the egocentric and allocentric distances were both matched for near and far spaces.

### Design and procedure

The color-label matching task was a 2 (association: self vs. stranger) × 2 (matching: matched vs. not matched) within-subjects design, divided into a training stage and a matching stage. First, in the training stage, the participants were required to code colored forks (black or white) as self or stranger. Specifically, the participants were told, “you are represented by a black fork, and a stranger is represented by a white fork”. After that, the participants had to judge whether the color-label pairings were correct in the matching stage. Specifically, each trial started with the presentation of a central fixation cross for 500 ms. Next, a pairing of color and label (you or stranger) was presented for 100 ms. The participants had to determine whether the color was correctly assigned to the person as accurately and quickly as possible within the timeframe (ranging from 800 to 1200 ms). The feedback (e.g., correct, wrong, or too slow) was presented for 500 ms at the end of each trial. The participants were required to perform 240 trials over three blocks, and they were informed of their overall accuracy at the end of the block. All the participants were explicitly informed that they could only conduct the spatial reference frame judgment tasks when their overall accuracy was higher than 90%. The color-label matching task served as training to familiarize the participants with the color-label associations. All the pairing conditions were counterbalanced across participants.

The spatial reference frame judgment task was a 2 (spatial domains: near vs. far) × 2 (reference frame judgment tasks: allocentric vs. egocentric) × 2 (groups: self-association vs. stranger-association) hybrid design. This task had two stages. First, the participants were randomly assigned to either the self-association group or stranger-association group. The self-association group was required to judge the position of the self-associated fork, and the stranger-association group was required to judge the position of the stranger-associated fork. Both groups were provided with verbal instruction that was the same as that provided in the color-label matching task (e.g., you are the white fork, the stranger is represented by the black fork). In the egocentric judgment task, each trial started with the presentation of instructions for 3000 ms. Afterward, the stimulus (a colored fork intersecting an orange plate) was presented for 150 ms. The participants had to determine whether the self- or stranger-association fork was on the left or right side of the midline of their body by pressing the response button with the right index finger or middle finger as accurately and quickly as possible within 1500 ms. The duration of each trial was 1650 ms with a 250 ms interstimulus interval. In the allocentric judgment task, the participants were required to determine whether the fork was on the left or right side of the midline of the plate. Except for the task instruction, the procedure of the allocentric representation task was the same as that of the egocentric judgment task. The participants needed to complete 384 trials of egocentric and allocentric judgment tasks in near and far spaces. Presentation order was counterbalanced across stimuli, tasks, spatial locations, and groups. In addition, the visual angle between the near space and far space was counterbalanced across participants. To ensure that the midline of the participants was aligned with the midline of the monitor, the participants’ head position was stabilized using a chin rest throughout the experiment.

### MRI acquisition

The participants were scanned on a 3.0 T Magnetom Prisma scanner with a commercial 64-element sensitivity encoding head coil array. For each participant, T1-weighted volumes were acquired using a magnetization-prepared gradient echo (MPRAGE) in an MRI room (number of layers = 36; slice thickness = 1 mm; scanning time = 5 min; FOV = 256 × 256 mm; scanning matrix size: 256 × 256 × 256; TR/TE = 2300/2.34 ms; flip angle = 8°).

### T1‑weighted image preprocessing and processing

The original T1-weighted images were converted from the DICOM format to the NIfTI format using MRIcroN software (dcm2niigui toolbox; https://www.nitrc.org/projects/mricron). After that, the oriented and neck-cut T1-weighted images were processed to obtain cortical thickness measures using FreeSurfer 6.0 software. FreeSurfer provides a full processing stream for T1-weighted MR images, including the removal of nonbrain tissue, automated Talairach transform computation, intensity normalization, skull stripping, white matter segmentation, filling and cutting, smoothing, inflation, spherical registration, and cortical parcellation statistics (https://surfer.nmr.mgh.harvard.edu/fswiki).

Brain structure measurements for cortical thickness were obtained using the semiautomated segmentation tool FreeSurfer. Cortical thickness maps were created using both signal intensity and continuity information from the 3D volume of magnetic resonance images, where the thickness was calculated as the closest distance from a pial to the white matter boundary at each vertex^70,71^. To avoid the misregistration of gray and white matter voxels, the quality of processed volumes was visually checked slice by slice using the FreeView toolbox before extracting the cortical thickness of the ROIs. Thickness maps were spatially smoothed using a Gaussian kernel with a half maximum width of 10 mm. The maps were then averaged across the participants using a spherical aligning method for cortical folding patterns. All operations to calculate cortical thickness were performed using code commands (https://surfer.nmr.mgh.harvard.edu/fswiki/FreeSurferCommands). Because the vmPFC acts as an ROI, the cortical thickness of the vmPFC in the medial orbitofrontal cortex (OFC), rostral and caudal anterior cingulate cortex (ACC), insular cortex, and frontal pole cortex (FPC) of the left and right hemispheres were extracted^72–74^.

## References

[1] Chan E, Baumann O, Bellgrove MA, Mattingley JB (2013). Extrinsic reference frames modify the neural substrates of object-location representations. Neuropsychologia.

[2] Arzy S, Schacter DL (2019). Self-agency and self-ownership in cognitive mapping. Trends Cogn. Sci..

[3] Colombo D, Serino S, Tuena C, Pedroli E, Dakanalis A, Cipresso P, Riva G (2017). Egocentric and allocentric spatial reference frames in aging: A systematic review. Neurosci. Biobehav. Rev..

[4] Chen Q, Weidner R, Weiss PH, Marshall JC, Fink GR (2012). Neural interaction between spatial domain and spatial reference frame in parietal-occipital junction. J. Cogn. Neurosci..

[5] Lane AR, Ball K, Smith DT, Schenk T, Ellison A (2013). Near and far space: Understanding the neural mechanisms of spatial attention. Hum. Brain Mapp..

[6] Lane AR, Ball K, Ellison A (2015). Dissociating the neural mechanisms of distance and spatial reference frames. Neuropsychologia.

[7] Wang A, Li Y, Zhang M, Chen Q (2016). The role of parieto-occipital junction in the interaction between dorsal and ventral streams in disparity-defined near and far space processing. PLoS One.

[8] Berti A, Frassinetti F (2000). When far becomes near: Remapping of space by tool use. J. Cogn. Neurosci..

[9] Zanini A, Patané I, Blini E, Salemme R, Koun E, Farnè A, Brozzoli C (2021). Peripersonal and reaching space differ: Evidence from their spatial extent and multisensory facilitation pattern. Psychon. Bull. Rev..

[10] Goodale MA, Haffenden A (1998). Frames of reference for perception and action in the human visual system. Neurosci. Biobehav. Rev..

[11] Goodale MA, Milner AD (1992). Separate visual pathways for perception and action. Trends Neurosci..

[12] Bernardino I, Mouga S, Castelo-Branco M, van Asselen M (2013). Egocentric and allocentric spatial representations in Williams syndrome. J. Int. Neuropsychol. Soc..

[13] Medina J, Kannan V, Pawlak MA, Kleinman JT, Newhart M, Davis C, Hillis AE (2009). Neural substrates of visuospatial processing in distinct reference frames: Evidence from unilateral spatial neglect. J. Cogn. Neurosci..

[14] Rolls ET (1999). Spatial view cells and the representation of place in the primate hippocampus. Hippocampus.

[15] Weiss PH, Marshall JC, Zilles K, Fink GR (2003). Are action and perception in near and far space additive or interactive factors?. Neuroimage.

[16] Andersen RA, Buneo CA (2002). Intentional maps in posterior parietal cortex. Annu. Rev. Neurosci..

[17] Bruno N (2001). When does action resist visual illusions?. Trends Cogn. Sci..

[18] Jiang YV, Swallow KM (2013). Spatial reference frame of incidentally learned attention. Cognition.

[19] Ruotolo F, Ruggiero G, Raemaekers M, Iachini T, Van der Ham IJM, Fracasso A, Postma A (2019). Neural correlates of egocentric and allocentric frames of reference combined with metric and non-metric spatial relations. Neuroscience.

[20] Ruggiero G, Ruotolo F, Orti R, Rauso B, Iachini T (2021). Egocentric metric representations in peripersonal space: A bridge between motor resources and spatial memory. Br. J. Psychol..

[21] Wang A, Shen L, Chi Y, Liu X, Chen Q, Zhang M (2016). Interaction between spatial domain and spatial reference frame in deaf and hearing populations. Acta Psychol. Sin..

[22] Quinlan DJ, Culham JC (2007). fMRI reveals a preference for near viewing in the human parieto-occipital cortex. Neuroimage.

[23] Gusnard DA, Raichle ME (2001). Searching for a baseline: Functional imaging and the resting human brain. Nat. Rev. Neurosci..

[24] Fox MD, Snyder AZ, Vincent JL, Corbetta M, Van Essen DC, Raichle ME (2005). The human brain is intrinsically organized into dynamic, anticorrelated functional networks. Proc. Natl. Acad. Sci..

[25] Corbetta M, Patel G, Shulman GL (2008). The reorienting system of the human brain: From environment to theory of mind. Neuron.

[26] Qin P, Northoff G (2011). How is our self related to midline regions and the default-mode network?. Neuroimage.

[27] Cosman JD, Vecera SP (2010). Attention affects visual perceptual processing near the hand. Psychol. Sci..

[28] Klein SB (2012). A role for self-referential processing in tasks requiring participants to imagine survival on the savannah. J. Exp. Psychol. Learn. Mem. Cogn..

[29] Spence C, Parise C (2010). Prior-entry: A review. Conscious. Cogn..

[30] Abrams RA, Davoli CC, Du F, Knapp WH, Paull D (2008). Altered vision near the hands. Cognition.

[31] Schäfer S, Frings C (2019). Searching for the inner self: Evidence against a direct dependence of the self-prioritization effect on the ventro-medial prefrontal cortex. Exp. Brain Res..

[32] Schäfer S, Wentura D, Frings C (2017). Distinctiveness effects in self-prioritization. Vis. Cogn..

[33] Sui J, Rotshtein P, Humphreys GW (2013). Coupling social attention to the self forms a network for personal significance. Proc. Natl. Acad. Sci..

[34] Frings C, Wentura D (2014). Self-prioritization processes in action and perception. J. Exp. Psychol. Hum. Percept. Perform..

[35] Bortolon C, Raffard S (2018). Self-face advantage over familiar and unfamiliar faces: A three-level meta-analytic approach. Psychon. Bull. Rev..

[36] Caughey S, Falbén JK, Tsamadi D, Persson LM, Golubickis M, Macrae CN (2020). Self-prioritization during stimulus processing is not obligatory. Psychol. Res..

[37] Alexopoulos T, Muller D, Ric F, Marendaz C (2012). I, me, mine: Automatic attentional capture by self-related stimuli. Eur. J. Soc. Psychol..

[38] Hu C, Di X, Eickhoff SB, Zhang M, Peng K, Guo H, Sui J (2016). Distinct and common aspects of physical and psychological self-representation in the brain: A meta-analysis of self-bias in facial and self-referential judgements. Neurosci. Biobehav. Rev..

[39] Cunningham SJ, Brebner JL, Quinn F, Turk DJ (2014). The self-reference effect on memory in early childhood. Child Dev..

[40] Sui J, He X, Humphreys GW (2012). Perceptual effects of social salience: Evidence from self-prioritization effects on perceptual matching. J. Exp. Psychol. Hum. Percept. Perform..

[41] Humphreys GW, Sui J (2015). The salient self: Social saliency effects based on self-bias. J. Cogn. Psychol..

[42] Humphreys GW, Sui J (2016). Attentional control and the self: The Self-Attention Network (SAN). Cogn. Neurosci..

[43] Sun Y, Fuentes LJ, Humphreys GW, Sui J (2016). Try to see it my way: Embodied perspective enhances self and friend-biases in perceptual matching. Cognition.

[44] Cunningham SJ, Turk DJ (2017). A review of self-processing biases in cognition. Q. J. Exp. Psychol..

[45] Yin S, Sui J, Chiu YC, Chen A, Egner T (2019). Automatic prioritization of self-referential stimuli in working memory. Psychol. Sci..

[46] Yin S, Bi T, Chen A, Egner T (2021). Ventromedial prefrontal cortex drives the prioritization of self-associated stimuli in working memory. J. Neurosci..

[47] Schäfer S, Wentura D, Frings C (2015). Self-prioritization beyond perception. Exp. Psychol..

[48] Chen Q, Weidner R, Vossel S, Weiss PH, Fink GR (2012). Neural mechanisms of attentional reorienting in three-dimensional space. J. Neurosci..

[49] Schäfer S, Wesslein AK, Spence C, Frings C (2021). When self-prioritization crosses the senses: Crossmodal self-prioritization demonstrated between vision and touch. Br. J. Psychol..

[50] McPhee AM, Constable MD, Saccone EJ, Welsh TN (2021). The influence of location, ownership, and the presence of a coactor on the processing of objects. Can. J. Exp. Psychol..

[51] Kurczek J, Wechsler E, Ahuja S, Jensen U, Cohen NJ, Tranel D, Duff M (2015). Differential contributions of hippocampus and medial prefrontal cortex to self-projection and self-referential processing. Neuropsychologia.

[52] Lieberman MD, Straccia MA, Meyer ML, Du M, Tan KM (2019). Social, self, (situational), and affective processes in medial prefrontal cortex (MPFC): Causal, multivariate, and reverse inference evidence. Neurosci. Biobehav. Rev..

[53] Philippi CL, Duff MC, Denburg NL, Tranel D, Rudrauf D (2012). Medial PFC damage abolishes the self-reference effect. J. Cogn. Neurosci..

[54] Qin P, Wang M, Northoff G (2020). Linking bodily, environmental and mental states in the self—A three-level model based on a meta-analysis. Neurosci. Biobehav. Rev..

[55] Meyer ML, Lieberman MD (2018). Why people are always thinking about themselves: Medial prefrontal cortex activity during rest primes self-referential processing. J. Cogn. Neurosci..

[56] Vogeley K, Fink GR (2003). Neural correlates of the first-person-perspective. Trends Cogn. Sci..

[57] Vogeley K, May M, Ritzl A, Falkai P, Zilles K, Fink GR (2004). Neural correlates of first-person perspective as one constituent of human self-consciousness. J. Cogn. Neurosci..

[58] Buss D (2015). Evolutionary Psychology: The New Science of the Mind.

[59] Klein SB, Cosmides L, Tooby J, Chance S (2002). Decisions and the evolution of memory: Multiple systems, multiple functions. Psychol. Rev..

[60] Desebrock C, Sui J, Spence C (2018). Self-reference in action: Arm-movement responses are enhanced in perceptual matching. Acta Physiol. (Oxf).

[61] Suh J, Abrams RA (2015). Reduced object-based perception in the near-hand space. Exp. Brain Res..

[62] Denny BT, Kober H, Wager TD, Ochsner KN (2012). A meta-analysis of functional neuroimaging studies of self-and other judgments reveals a spatial gradient for mentalizing in medial prefrontal cortex. J. Cogn. Neurosci..

[63] Erpelding N, Moayedi M, Davis KD (2012). Cortical thickness correlates of pain and temperature sensitivity. Pain.

[64] Fleming KA, Heintzelman SJ, Bartholow BD (2016). Specifying associations between conscientiousness and executive functioning: Mental set shifting, not prepotent response inhibition or working memory updating. J. Pers..

[65] Kühn S, Schubert F, Gallinat J (2011). Structural correlates of trait anxiety: Reduced thickness in medial orbitofrontal cortex accompanied by volume increase in nucleus accumbens. J. Affect. Disord..

[66] Liem F, Mérillat S, Bezzola L, Hirsiger S, Philipp M, Madhyastha T, Jäncke L (2015). Reliability and statistical power analysis of cortical and subcortical FreeSurfer metrics in a large sample of healthy elderly. Neuroimage.

[67] Faul F, Erdfelder E, Buchner A, Lang AG (2009). Statistical power analyses using G* Power 3.1: Tests for correlation and regression analyses. Behav. Res. Methods.

[68] Faul F, Erdfelder E, Lang AG, Buchner A (2007). G* Power 3: A flexible statistical power analysis program for the social, behavioral, and biomedical sciences. Behav. Res. Methods.

[69] Cohen J (2013). Statistical Power Analysis for the Behavioral Sciences.

[70] Dale AM, Fischl B, Sereno MI (1999). Cortical surface-based analysis: I. Segmentation and surface reconstruction. Neuroimage.

[71] Gautam P, Anstey KJ, Wen W, Sachdev PS, Cherbuin N (2015). Cortical gyrification and its relationships with cortical volume, cortical thickness, and cognitive performance in healthy mid-life adults. Behav. Brain Res..

[72] Striedter GF (2005). Principles of Brain Evolution.

[73] Murray EA, Wise SP, Graham KS (2017). The Evolution of Memory Systems: Ancestors, Anatomy, and Adaptations.

[74] Desikan RS, Ségonne F, Fischl B, Quinn BT, Dickerson BC, Blacker D, Dale AM, Maguire RP, Hyman BT, Alber MS, Killiany RJ (2006). An automated labeling system for subdividing the human cerebral cortex on MRI scans into gyral based regions of interest. Neuroimage.

